# Adenosine A_2A_ receptors control synaptic remodeling in the adult brain

**DOI:** 10.1038/s41598-022-18884-4

**Published:** 2022-08-29

**Authors:** Xinli Xu, Rui O. Beleza, Francisco Q. Gonçalves, Sergio Valbuena, Sofia Alçada-Morais, Nélio Gonçalves, Joana Magalhães, João M. M. Rocha, Sofia Ferreira, Ana S. G. Figueira, Juan Lerma, Rodrigo A. Cunha, Ricardo J. Rodrigues, Joana M. Marques

**Affiliations:** 1 CNC-Center for Neuroscience and Cell Biology, Faculdade de Medicina Polo I, University of Coimbra, Rua Larga, Piso 1, 3004-504 Coimbra, Portugal; 2 Institute of Interdisciplinary Research, University of Coimbra, 3030-789 Coimbra, Portugal; 3 Instituto de Neurociencias de Alicante, CSIC-UMH, 03550 San Juan de Alicante, Spain; 4 Faculty of Medicine, University of Coimbra, 3004-504 Coimbra, Portugal

**Keywords:** Neuroscience, Cellular neuroscience, Development of the nervous system, Diseases of the nervous system

## Abstract

The molecular mechanisms underlying circuit re-wiring in the mature brain remains ill-defined. An eloquent example of adult circuit remodelling is the hippocampal mossy fiber (MF) sprouting found in diseases such as temporal lobe epilepsy. The molecular determinants underlying this retrograde re-wiring remain unclear. This may involve signaling system(s) controlling axon specification/growth during neurodevelopment reactivated during epileptogenesis. Since adenosine A_2A_ receptors (A_2A_R) control axon formation/outgrowth and synapse stabilization during development, we now examined the contribution of A_2A_R to MF sprouting. A_2A_R blockade significantly attenuated status epilepticus(SE)-induced MF sprouting in a rat pilocarpine model. This involves A_2A_R located in dentate granule cells since their knockdown selectively in dentate granule cells reduced MF sprouting, most likely through the ability of A_2A_R to induce the formation/outgrowth of abnormal secondary axons found in rat hippocampal neurons. These A_2A_R should be activated by extracellular ATP-derived adenosine since a similar prevention/attenuation of SE-induced hippocampal MF sprouting was observed in CD73 knockout mice. These findings demonstrate that A_2A_R contribute to epilepsy-related MF sprouting, most likely through the reactivation of the ability of A_2A_R to control axon formation/outgrowth observed during neurodevelopment. These results frame the CD73-A_2A_R axis as a regulator of circuit remodeling in the mature brain.

## Introduction

The current knowledge on the mechanisms driving circuit re-wiring in the mature central nervous system is limited. Yet, this knowledge is fundamental to understand and correctly promote nerve regeneration, control stem cell grafting or adult-born neurons integration or prevent pathological circuit remodeling. A very well-established example of such re-wiring processes in the adult brain is the hippocampal mossy fiber (MF) sprouting found in patients and in animal models of temporal lobe epilepsy (TLE)^1,2^.

The retrodirective growth of MF into the inner molecular layer of the dentate gyrus (DG) ^3,4^ seems to be due to a dysregulation of guidance cues involved in the establishment of normal MF connectivity during development^5–8^ but the underlying molecular determinants are still unclear. BDNF has been associated to this initial axonal branching^9–11^ but other studies observed MF sprouting in the absence of BDNF^12^ or showed that BDNF is not sufficient to induce MF sprouting^13,14^. which critically requires abnormal circuit activity^15^.

One unexplored set of candidates to regulate mature circuit remodeling, in particular hippocampal MF sprouting, are purines; purines are released in an activity-dependent manner^16^ and can control neuronal migration and wiring during development, particularly through adenosine A_2A_ receptors (A_2A_R). ATP is preferentially released from MF terminals at high-frequency stimulations^17,18^ and we recently reported that A_2A_Rs control axon formation and outgrowth of migrating cortical projection neurons during embryogenesis^19^. being also involved in synaptic stabilization during development^20^. Besides, A_2A_Rs are located in hippocampal MF terminals^18^. contribute to synaptic failure and transient decrease in cell proliferation in the DG upon oxygen–glucose deprivation^21^. are active during epileptogenesis^18,22–24^ and their pharmacological blockade or genetic deletion prevent seizure-induced neurodegeneration^23^ and attenuate the progressive seizure severity in different models of kindling^23,25^.

In this study, we provide evidence that A_2A_Rs, in particular those located in dentate granule cells are activated by ATP-derived adenosine and contribute to status epilepticus (SE)-induced hippocampal MF sprouting, most likely by inducing axon formation and outgrowth. This demonstrates a reactivation of adenosine-induced A_2A_R-mediated control of brain wiring in the adult brain, impacting on circuit remodeling after SE.

## Results

### Pharmacological activation of A_2A_Rs induces the formation of secondary aberrant axons in cultured hippocampal neurons

**Figure 1:**
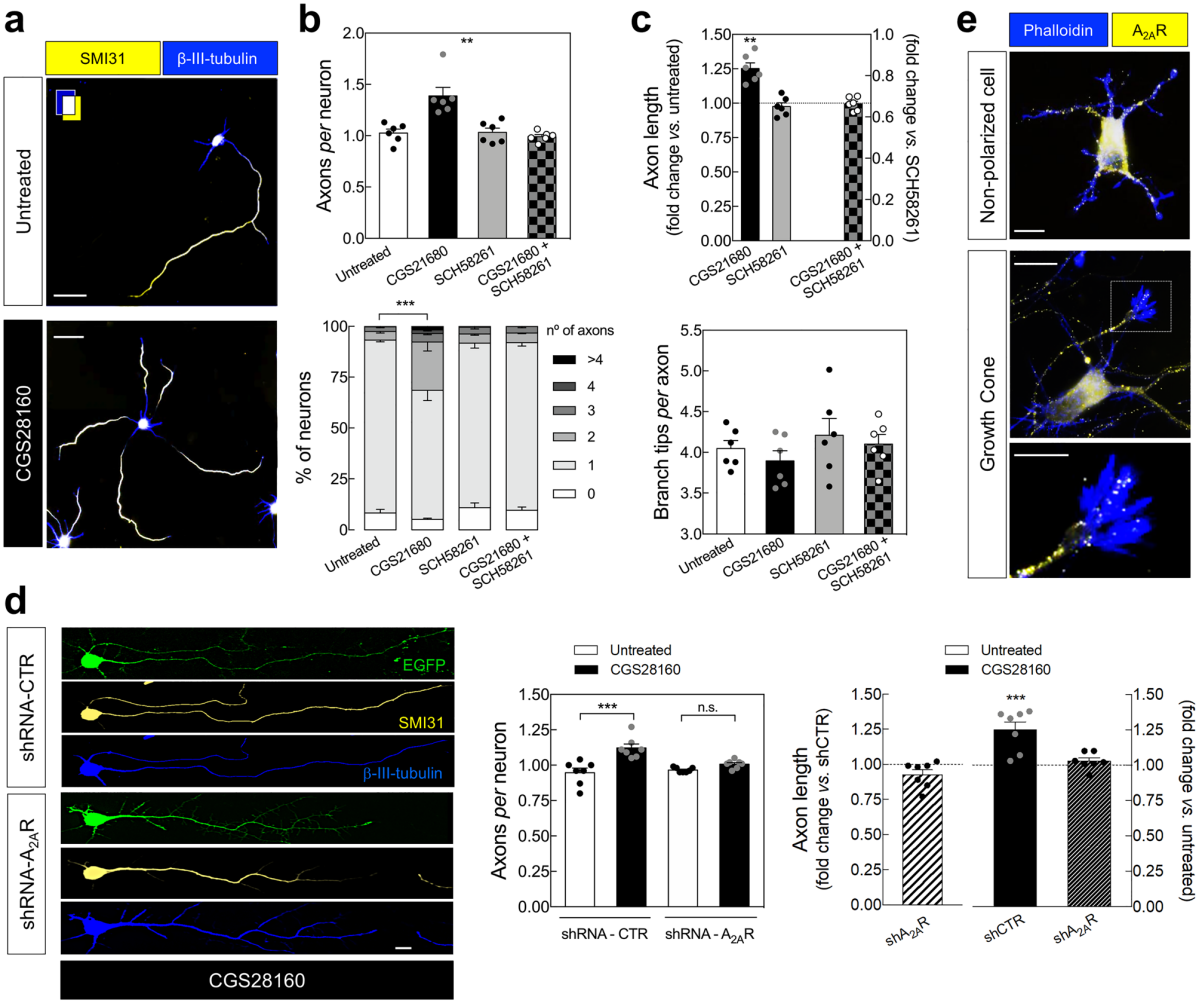
Pharmacological activation of A_2A_Rs induces the formation of abnormal secondary axons in cultured hippocampal neurons. (**a**) Representative images of E18 rat-derived hippocampal neurons cultured in the absence and in the presence of the selective agonist of A_2A_Rs, CGS21680 (30 nM), from DIV0, double immunolabeled with SMI31 (axonal marker; yellow) and βIII-tubulin (neuronal marker; blue) antibodies at DIV 3 (scale bar, 30 µm), showing that (**b**) the exposure to CGS21680 increased the number of axons *per* neuron (upper graph), reflecting an increased percentage of neurons with multiple axons (lower graph). The selective antagonist of A_2A_Rs, SCH58261 (50 nM), did not modify the average number of axons *per* neuron, but prevented the ability of CGS21680 to modify the number of axons *per* neuron. (**c**) CGS21680 increased axonal length (in those cells displaying more than one axon, it was counted the longest axon), whereas SCH58261 was devoid of effects. In the presence of SCH58261, CGS21680 did not modify axonal length. Neither CGS21680 nor SCH58261 significantly modified axonal branching. Data are mean ± SEM quantified from 6 independent cultures, analyzing a minimum of 100 cells *per* culture and condition. (**d**) In cells electroporated at DIV0 with either shRNA-Control (shCTR) or shRNA-A_2A_R (shA_2A_R) (EGFP^+^), CGS21680 increased the number of axons *per* neuron and axonal length in cells transfected with shCTR but not in cells transfected with shA_2A_R. Scale bar, 10 μm. Data are mean ± SEM quantified from 7 independent cultures, analyzing a minimum of 25 transfected cells *per* culture and condition. **P < 0.01 and ***P < 0.001. (**b** *upper*; **c** lower) one-way ANOVA with Dunnet’s *post-hoc* test; (**b** *lower*; **d** *left*) two-way ANOVA with Sidak’s *post-hoc* test; (**c** *upper*; **d** *right*) one-sample *t*-test *vs.* hypothetical value of 1. (**e**) Immunocytochemical analysis of A_2A_R immunoreactivity (yellow) in a non-polarized neuron and in an axonal growth cone labelled with phalloidin (blue). Scale bar for upper and middle image, 10 μm; scale bar for the lower image, 5 μm.

To start addressing if A_2A_Rs control axon formation and/or outgrowth of hippocampal neurons, as previously observed in cortical neurons^19^ we first evaluated the impact of the pharmacological manipulation of A_2A_Rs in axon formation/outgrowth of cultured E18-rat derived hippocampal neurons. We observed that the pharmacological activation of A_2A_Rs with the selective agonist CGS21680 (30 nM) from DIV0 caused an increase in the number of axons *per* neuron, reflecting an increase in the percentage of neurons with more than one axon, analyzed at DIV3 (Fig. 1a,b). CGS21680 also increased axonal length without affecting axonal branching (Fig. 1c). The selective antagonist of A_2A_R, SCH58261 (50 nM), did not significantly modify neither axon formation nor axonal elongation (Fig. 1b,c), showing that the tonic activation of A_2A_Rs by endogenous adenosine is absent or does not affect axon formation/outgrowth. Accordingly, the knockdown of A_2A_Rs, using a validated short-hairpin RNA expressing a construct against A_2A_R (shRNA-A_2A_R), neither significantly modified the number of axons *per* neuron nor axon elongation, analyzed at DIV3 (Fig. 1d and Supplementary Figs. 1, 2). The increase in the number of axons *per* neuron and axon length caused by CGS21680 are mediated by the activation of A_2A_Rs since it was not observed in the presence of SCH58261 (Fig. 1b,c) and was observed in cells electroporated with a short-hairpin RNA expressing a construct against a non-targeting control (shRNA-Control) but not in cells expressing shRNA-A_2A_R (Fig. 1d).

These results show that the pharmacological activation of A_2A_Rs promotes axonal elongation and induces the formation of secondary abnormal axons in developing rat hippocampal neurons. Accordingly, we observed an immunoreactivity for A_2A_R in non-polarized cells as well as in axonal growth cones (Fig. 1e). These findings in hippocampal neurons converge with a previous report in cortical neurons^19^, suggesting that A_2A_Rs control axogenesis in different neuronal populations.

### A_2A_Rs contribute to status epilepticus-induced hippocampal mossy fiber sprouting

**Figure 2:**
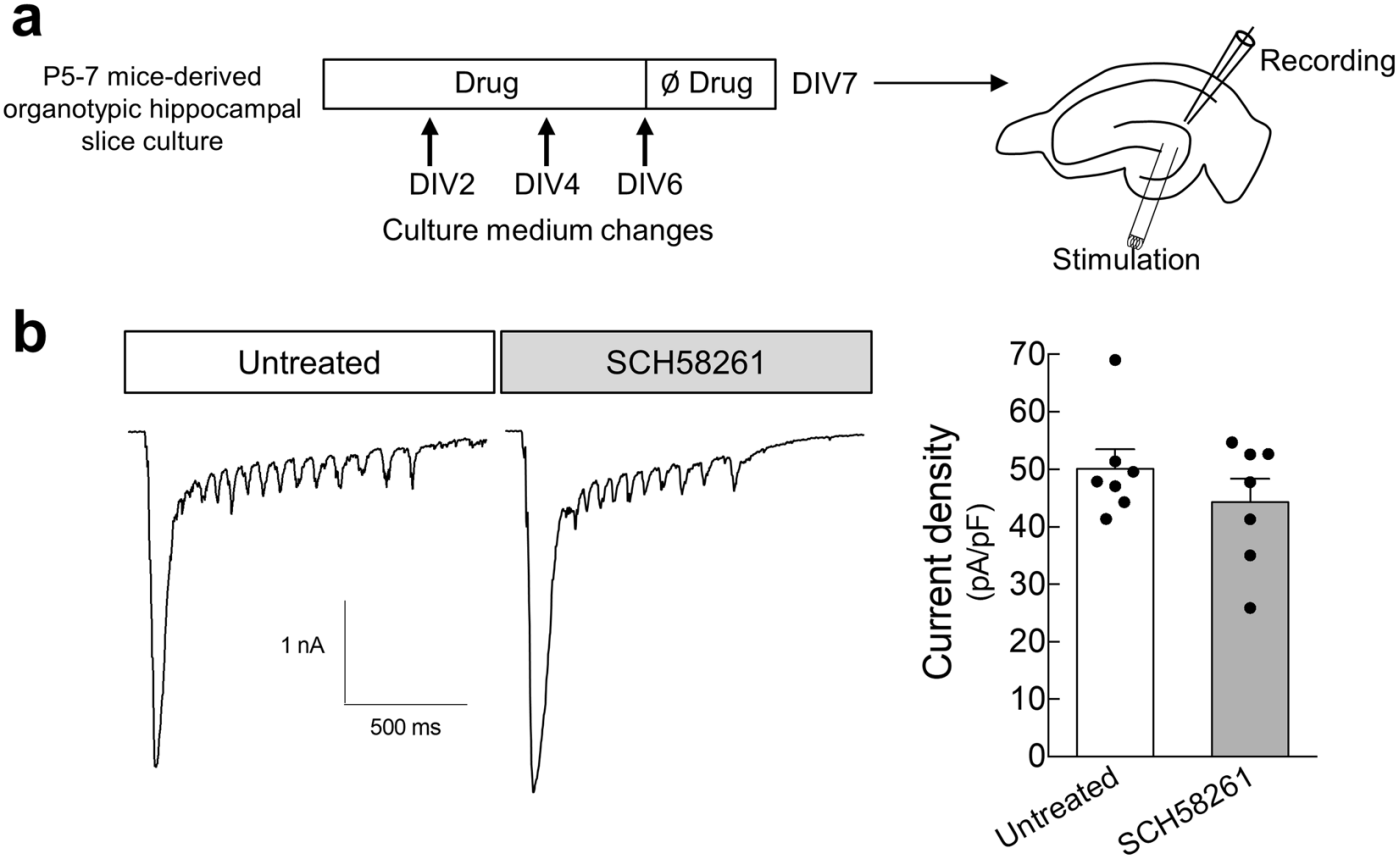
Pharmacological blockade of A_2A_Rs does not modify the spontaneously-formed mossy fiber recurrent pathways in hippocampal organotypic slices. (**a**) P5-7 mice-derived hippocampal organotypic slices were cultured either in the absence or in the presence of SCH58261 (50 nM) until DIV 6, and at DIV 7 synaptic currents at dentate granule cells elicited by stimulation of mossy fibers at the hilus were recorded by whole-cell patch-clamp. (**b**) SCH58261 did not significantly modify the density of synaptic current in dentate granule cells upon stimulation at the hilus, as depicted in the representative traces and summarized in the histogram. Data are mean ± SEM quantified from 7 independent cultures, recording 6–13 cells *per* culture *per* condition. *P* > 0.05 unpaired *t*-test.

Hippocampal organotypic slices display spontaneous MF sprouting in the molecular layer of the DG^26–28^ supporting an enhanced excitation of dentate granule cells^29^. In hippocampal organotypic slices prepared from P5-7 mice, stimulation of MF at DIV7 elicited synaptic currents in dentate granule cells (Fig. 2). In slices cultured in the presence of the A_2A_R antagonist SCH58261 (50 nM), the current density was not significantly modified (Fig. 2), indicating that the formation of these recurrent excitatory inputs at the dentate granule cells in hippocampal organotypic slices is unrelated to the activation of A_2A_Rs by endogenous adenosine.

**Figure 3:**
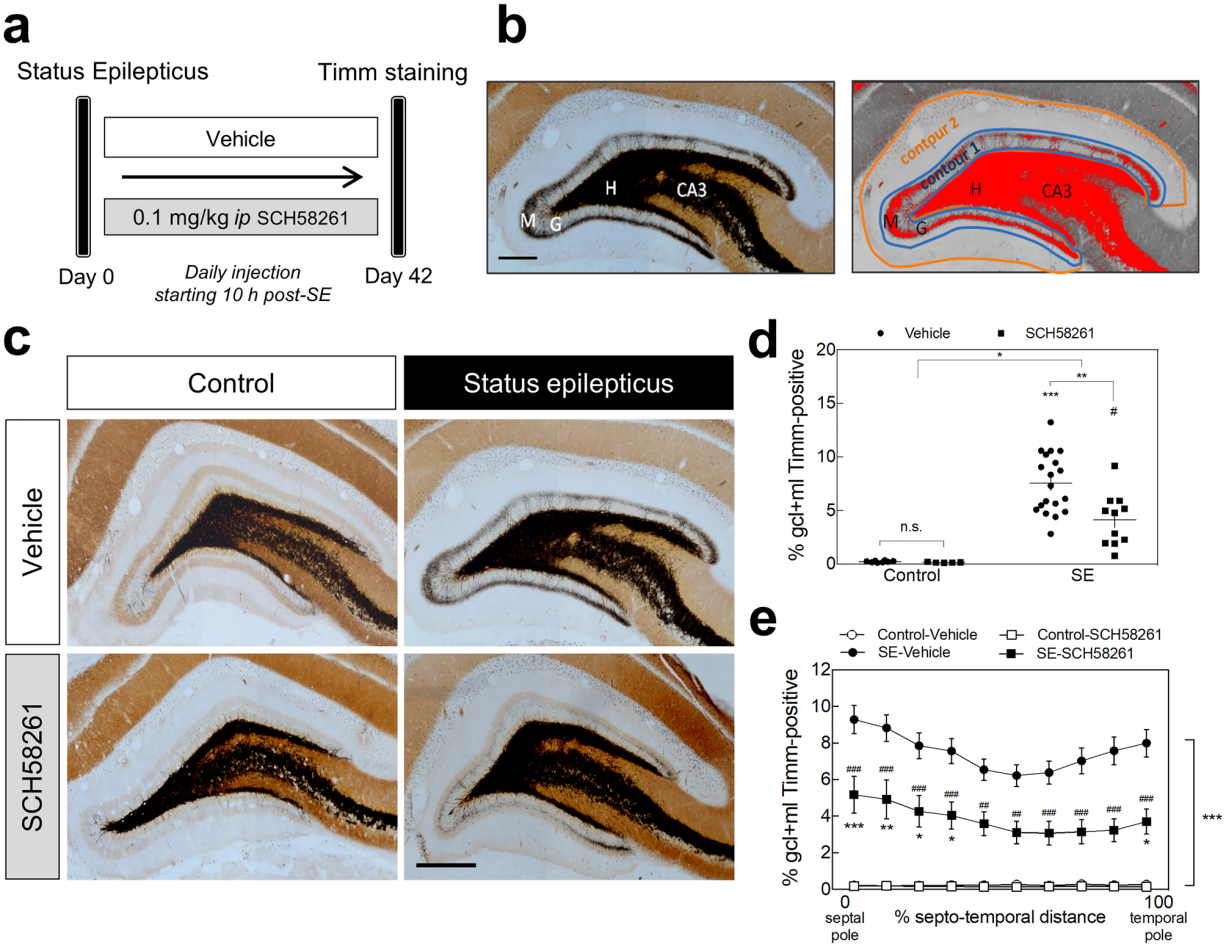
Pharmacological blockade of A_2A_Rs reduces status epilepticus-induced mossy fiber sprouting in the rat hippocampus. (**a**) Six/seven weeks old rats were either injected with saline (Control group) or with pilocarpine (Status epilepticus group—SE), 30 min after administration of scopolamine methylbromide (2 mg/kg, *i.p.*). Only animals reaching at least stage 4 seizures were considered in this study. Convulsions were suppressed with diazepam (10 mg/kg, *i.p.*) 2 h after the occurrence of the first stage 4 seizure. The rats from each group were assigned into two subgroups: one subgroup was daily injected with the selective antagonist of A_2A_Rs, SCH58261 (0.1 mg/kg *i.p.*), and the other with vehicle (0.2% Tween-20 in saline), starting 10 h after the onset of status epilepticus (SE). (**b**) Forty-two days later, hippocampal mossy fiber (MF) sprouting was analyzed by Timm staining as previously described^102,103^. The extent of MF sprouting was assessed by estimating the Timm-positive area (adjusting a darkness threshold setting until the selected area in red matched the black Timm-positive area of the same section)/volume measured in granule cell layer (GCL) + inner molecular layer (IML) (Contour 1) over the total volume/area of the GCL + molecular layer (ML) (Contour 2). H-Hilus; G-granule cell layer; M-molecular layer. (**c**) Rats that had developed SE displayed a dense Timm staining in the IML of the DG in comparison with Control animals. This staining was significantly lower in SE rats daily injected with SCH58261 as depicted in the representative images (scale bar, 300 μm) and (**d**) quantitatively summarized in the histogram. (**e**) The reduction in MF sprouting in SCH58261-treated SE rats was observed along the entire septotemporal axis of the DG. Control-Vehicle, *n* = 8; Control-SCH58261, *n* = 5; SE-Vehicle, *n* = 20; SE-SCH58261, *n* = 11. The data are mean ± SEM of the percentage of the GCL + ML displaying Timm staining. In (**d**), two-way ANOVA with Sidak’s *post-hoc* test; *P < 0.05, **P < 0.01, ***P < 0.001 and ^#^*P* < 0.05 for SE-SCH58261 *vs.* Control-SCH58261. In (**e**), two-way ANOVA with Sidak’s *post-hoc* test; *P < 0.05, **P < 0.01 and ***P < 0.0001 for SE *vs.* Control either with vehicle or SCH58261; ^##^*P* < 0.01 and ^###^*P* < 0.001 for SCH58261 *vs.* vehicle with or without SE.

Next, we evaluated the contribution of A_2A_Rs to hippocampal MF sprouting induced by SE. We resorted to the pilocarpine-induced model of SE in rats, which triggers a robust hippocampal MF sprouting within weeks to months after SE^30^. Forty-two days after SE induction, a positive Timm staining demonstrated MF sprouting in the granule cell and molecular layers of the DG (Fig. 3a,b). Only pilocarpine-treated animals reaching at least stage 4 seizures were considered in this study. To appraise the contribution of A_2A_Rs, rats were injected daily with SCH58261 (0.1 mg/kg, *i.p.*) or vehicle, beginning 10 h after the onset of SE (Fig. 3a). The extent of MF sprouting was assessed by estimating the percentage of the granule cell layer plus molecular layer that presented Timm staining (Fig. 3b). In control rats that had not experienced SE, non-significant levels of Timm staining were observed in the granule cell layer and inner molecular layer either in vehicle or SCH58261-treated rats (Fig. 3c,d). In contrast, animals that had developed SE and had been administered with vehicle for 42 days, displayed a significantly denser Timm staining in the entire granule cell layer and inner molecular layer of the DG (*P* < 0.0001). In rats administered with SCH58261, SE still induced a significantly higher Timm staining than in control (*P* = 0.013 SE-SCH58261 *vs.* Control-SCH58261), but considerably lower than the observed in vehicle-injected rats subjected to SE (*P* = 0.039 for SE *vs.* SCH58261 interaction; *P* = 0.004 for SE-vehicle *vs.* SE-SCH58261) (Fig. 3c,d). SE-induced MF sprouting was observed along the entire septotemporal axis (Fig. 3e) both in control and, albeit with significantly lower levels, in SCH58261-treated animals (Fig. 3e).

These results indicate that A_2A_Rs contribute to SE-induced hippocampal MF sprouting.

### A_2A_Rs expressed by dentate granule cell neurons contribute to mossy fiber sprouting

If the contribution of A_2A_Rs to SE-induced hippocampal MF sprouting is due to a direct control of axon formation/outgrowth, as observed in cultured hippocampal neurons, A_2A_Rs expressed in dentate granule cells should mediate this effect. Thus, we evaluated the impact of knocking down A_2A_Rs in dentate granule cells using lentivectors encoding the reporter gene EGFP and either shRNA-A_2A_R or shRNA-Control (Refs 31, 32 and Supplementary Figs. 1, 2).

**Figure 4:**
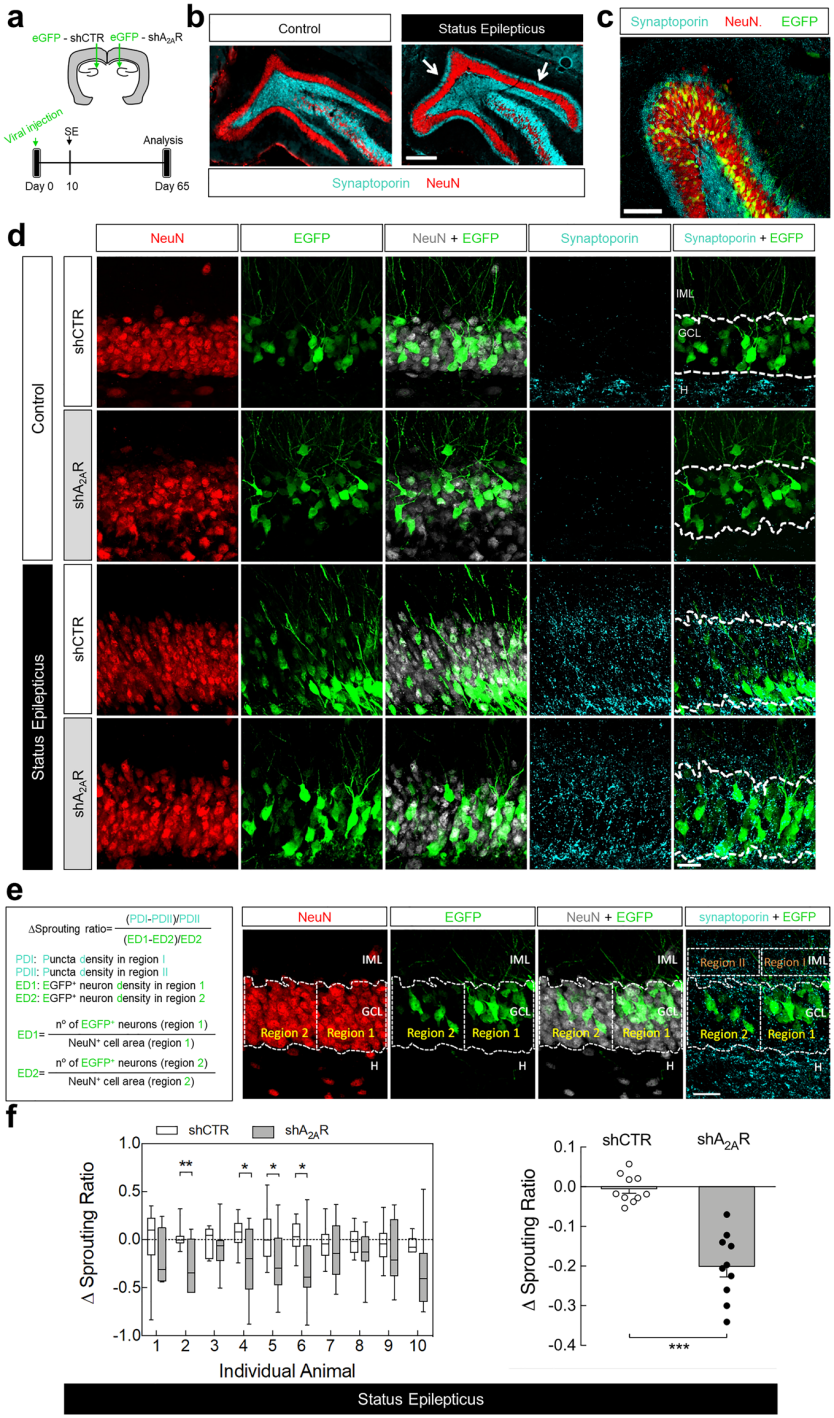
Knockdown of A_2A_Rs in dentate granule cells reduces status epilepticus-induced hippocampal mossy fiber sprouting. (**a**) Lentivirus encoding EGFP and shRNA-Control (shCTR) or shRNA-A_2A_R (shA_2A_R) were injected into the hippocampal dentate gyrus (DG) in the left or in the right hemisphere. After 10 days, rats were injected with saline (Control group) or pilocarpine (Status epilepticus group). All rats were administered with scopolamine methylbromide (2 mg/kg, *i.p.*) 30 min before. Only animals reaching at least stage 4 seizures were considered. (**b**) After 55 days, mossy fiber (MF) sprouting was assessed by immunohistochemical analysis of synaptoporin (MF terminals marker). Representative images of the immunolabelling of synaptoporin and neuronal nuclei (NeuN) showing that in rats that experienced status epilepticus (SE) it was observed synaptoporin immunoreactivity in the inner molecular layer (IML) of the DG (white arrows), which was not observed in control rats. Scale bar, 300 μm. (**c**) Representative image showing a DG segment infected with lentivirus encoding shCTR from a rat that experienced SE, immunolabelled with synaptoporin and NeuN and displaying EGFP^+^-dentate granule cells. Scale bar, 100 µm. (**d**) Schematic illustration of the quantitative method used to analyse the extent of MF sprouting. A Δsprouting ratio for each acquired image with obviously different EGFP^+^-cell density in adjacent areas was calculated by measuring the incremental proportion of the synaptoporin (cyan) puncta density (PD2 to PD1) in the IML *vs.* the incremental proportion of EGFP^+^-neuron density (ED2 to ED1) in the granule cell layer (GCL), defined by NeuN immunoreactivity. (**e**) The brain sections from shCTR- or shRNA-A_2A_R-injected DG from control animals displayed no synaptoporin staining (cyan) in the IML. In SE group, shA_2A_R-injected DG sections displayed lower synaptoporin puncta densities in the IML region corresponding to the higher EGFP^+^-cell density region in GCL which was not observed in shCTR-injected DG. Scale bar, 25 μm. (**f**) Δsprouting ratio in shA_2A_R-injected DG and shCTR-injected DG in each individual animal that had experienced SE (*left*), supporting a significant lower Δsprouting in shA_2A_R-injected DG *vs.* shCTR-injected DG (right). The data are median and interquartile range (*left* graph) or mean ± SEM (*right* graph). *P < 0.05 and ***P < 0.001, paired *t*-test. H-Hilus.

Each rat was stereotaxically injected into the left or right DG with either shRNA-Control or shRNA-A_2A_R and was allowed to recover for 10 days before SE induction (Fig. 4a). Only animals reaching at least stage 4 seizures were considered in this study, and they were sacrificed 56 days after SE for the evaluation of MF sprouting in the inner molecular layer. This was performed by immunohistochemical identification of sprouted MF puncta by immunolabelling synaptoporin (Fig. 4b), a synaptic vesicle membrane protein highly enriched in MF terminals^33–35^. The lentivectors used have been previously shown to have a limited spread within the brain parenchyma^36^ infecting only neurons^32^. Accordingly, EGFP^+^-cells were all endowed with NeuN^+^-immunolabelling (Fig. 4c).

Control animals showed no MF sprouting, evidenced by the lack of synaptoporin staining in the inner molecular layer, either in the DG injected with shRNA-Control or shRNA-A_2A_R (Fig. 4d). The rats that experienced SE displayed synaptoporin labelling in the granule cell and inner molecular layers (Fig. 4d). To estimate the impact of the knockdown of A_2A_Rs in dentate granule cells in MF sprouting, we determined a Δsprouting ratio to evaluate the correlation between the density of infected granule cells (EGFP^+^) and sprouted MF puncta density in the adjacent inner molecular layer for each image acquired from animals that had experienced SE (Fig. 4e). We found a negative correlation between the density of synaptoporin puncta in the inner molecular layer and granule cell layer and EGFP^+^-cell density in the adjacent granule cell layer region in shRNA-A_2A_R-injected DG, but not in shRNA-Control-injected DG (Fig. 4e,f). In the analysis of individual animals, the Δsprouting ratio of shRNA-A_2A_R-injected DG was significantly different from shRNA-Control-injected DG in 4 out of 10 animals (Fig. 4f left graph). An overall analysis of all the animals that developed SE revealed that in shRNA-Control-injected DG, the Δsprouting ratio was not different from zero, showing no impact of shRNA-Control in synaptoporin puncta density. In contrast, shRNA-A_2A_R-injected DG displayed a significantly negative Δsprouting ratio (*P* < 0.0001 *vs.* hypothetical value of 0) and statistically different from the Δsprouting ratio of shRNA-Control (*P* = 0.0001; paired *t*-test). These data show that the knockdown of A_2A_Rs in granule cells attenuates synaptoporin puncta density in the inner molecular layer and granule cell layer, demonstrating that A_2A_Rs located in dentate granule cells contribute to SE-induced MF sprouting.

### Impact of the pharmacological blockade of A_2A_Rs in SE-induced cell proliferation

MF sprouting into the inner molecular layer occurs in the absence of neurogenesis^37^. Yet, seizures induce the generation of new dentate granule cells from the subgranular zone^38–40^ and these post-SE born cells also contribute further to MF sprouting, in particular at later stages^41,42^. Indeed, cells born after SE display hilar basal dendrites and ectopic migration, but do not send axons into the inner molecular layer until 4 weeks after the insult ^41,42^ and only have a substantial contribution to MF sprouting 10 weeks after SE^41^.

**Figure 5:**
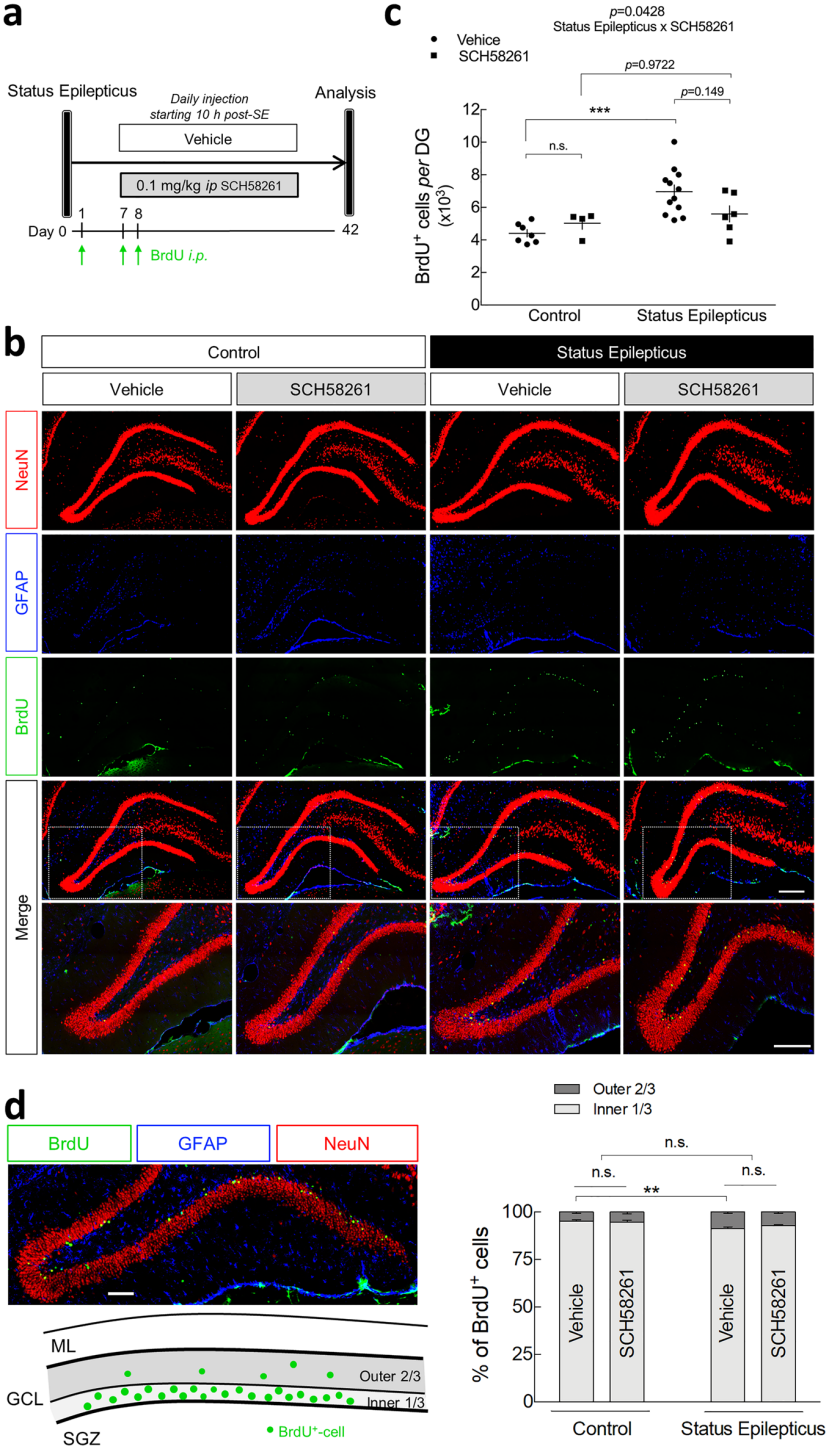
Impact of the pharmacological blockade of A_2A_Rs in status epilepticus-induced cell proliferation and migration of adult-born neurons. (**a**) Six/seven weeks old rats were injected either with saline (Control group) or with pilocarpine (Status Epilepticus group), 30 min after the administration of scopolamine methylbromide (2 mg/kg, *i.p.*). Only animals reaching at least stage 4 seizures were considered. Convulsions were suppressed with diazepam (10 mg/kg, *i.p.*) 2 h after the occurrence of the first stage 4 seizure. The rats from each group were assigned into two subgroups: one subgroup was daily injected with the selective antagonist of A_2A_R, SCH58261 (0.1 mg/kg *i.p.*), and the other with vehicle (0.2% Tween-20 in saline), starting 10 h after the onset of status epilepticus. All the animals were *i.p.* injected with BrdU at 24 h (200 mg/kg), 7 days (200 mg/kg) and 8 days (100 mg/kg) after status epilepticus. BrdU^+^-cells in the granule cell layer (GCL) *per* dentate gyrus (DG) was evaluated at 42 days post-status epilepticus by (**b**) immunohistochemical analysis of BrdU (green), NeuN (magenta) and GFAP (cyan). Scale bar, 300 μm. (**c**) Status epilepticus induced a significant increase in BrdU^+^-cells in the GCL *per* DG in vehicle-injected rats. SCH58261 significantly modified status epilepticus-induced increase in BrdU^+^-cells density (*P < 0.05 status epilepticus x SCH58261 interaction; two-way ANOVA). *Post-hoc* analysis revealed that status epilepticus did not significantly increase BrdU^+^-cells density in rats administered with SCH58261, but also there was no significant difference between status epilepticus-vehicle and status epilepticus-SCH58261. (**d**) Vehicle-injected rats that experienced status epilepticus displayed a higher relative percentage of BrdU^+^-cells in the outer two-thirds of the GCL in comparison with the Control group. No interaction was found between SCH58261 and status epilepticus factors (two-way ANOVA). The data are mean ± SEM of the number of the BrdU^+^-cells in GCL or the relative percentage of cells in the inner (1/3) and in the outer (2/3) parts of GCL. Control-Vehicle, *n* = 7; Control-SCH58261, *n* = 4; Status Epilepticus-Vehicle, *n* = 12; Status Epilepticus-SCH58261, *n* = 6. ML—molecular layer; GCL—granule cell layer; SGZ—subgranular zone. *P < 0.05, **P < 0.01 and ***P < 0.001, two-way ANOVA with Sidak’s test.

A_2A_Rs may promote adult neurogenesis particularly in pathological conditions^43,44^. Hence, we sought to determine if, in addition to the contribution of A_2A_Rs located in dentate granule cells born before SE, A_2A_Rs may also further promote hippocampal MF sprouting by contributing to the generation of newborn cells. To address this, we tested whether the daily administration of the selective A_2A_R antagonist SCH58261 (0.1 mg/kg, *i.p.*), beginning 10 h after the onset of SE, affected SE-induced cell proliferation in the granule cell layer. To label mitotically active cells, rats were injected with BrdU at 24 h (200 mg/kg, *i.p.*), 7 days (200 mg/kg, *i.p.*) and 8 days (100 mg/kg, *i.p.*) after SE (Fig. 5a), the latter two time points being within the period of significantly increased mitosis^37^. Consistent with previous reports^38,45,46^, quantitative analysis revealed a significant increase in the number of BrdU^+^-cells in the granule cell layer in SE rats treated with vehicle for 42 days in comparison with control rats (*P* < 0.001; Fig. 5b,c). The vast majority of BrdU^+^-cells were co-localized with the mature neuronal marker NeuN (Fig. 5b), in agreement with previous reports describing that the majority (~ 90%) of the newly generated cells within the granule cell layer after SE differentiate into neurons^38,45,46^. In control rats, SCH58261 administration per se did not significantly modify the density of BrdU^+^-cells in the granule cell layer, showing that A_2A_Rs do not contribute to basal adult cell proliferation originated from the subgranular zone. Regarding the impact of SCH58261 on SE-induced increase in BrdU^+^-cells in the DG, we found an interaction between SE and SCH58261 (*P* = 0.0428; two-way ANOVA). Yet, *post-hoc* analysis showed that SE-vehicle and SE-SCH58261 groups were not significantly different (*P* = 0.1492; Fig. 5c). On the other hand, in rats daily injected with SCH58261, SE did not induce a statistically significant increase in BrdU^+^-cells (*P* = 0.9722 for SE-SCH58261 *vs.* Control-SCH58261).

The newborn neurons generated in the subgranular zone migrate radially into the granule cell layer, mainly integrating functionally into the existing circuitry in the inner third of the granule cell layer^47,48^. Following an epileptogenic insult, there is a misplacement of a significant number of cells that migrate abnormally to the outer two-thirds of granule cell layer^38,40^. Accordingly, we now observed in control rats that most of the BrdU^+^-cells were located in the inner one-third of the granule cell layer and only a small portion of BrdU^+^-cells were located in the outer two-thirds of the granule cell layer (4.82 ± 0.73%; *n* = 7) (Fig. 5d). SCH58261 administration per se did not modify this pattern in control rats. However, SE rats treated with vehicle for 42 days displayed a significant increase in the relative percentage of BrdU^+^-cells in the outer two-thirds of the granule cell layer (8.66 ± 0.77%, *n* = 12), compared to controls (Fig. 5d). In contrast to the observed in SE-induced increase in BrdU^+^-cells density (Fig. 5c), SCH58261 administration did not modify the SE-induced spatial distribution of adult newborn cells in the granule cell layer (*P* = 0.3058 for SE x SCH58261 interaction; two-way ANOVA) (Fig. 5d). These data show that A_2A_Rs are not involved in SE-induced aberrant migration of adult-born cells within the granule cell layer.

### Status epilepticus-induced hippocampal mossy fiber sprouting is engaged by extracellular ATP-derived adenosine

**Figure 6:**
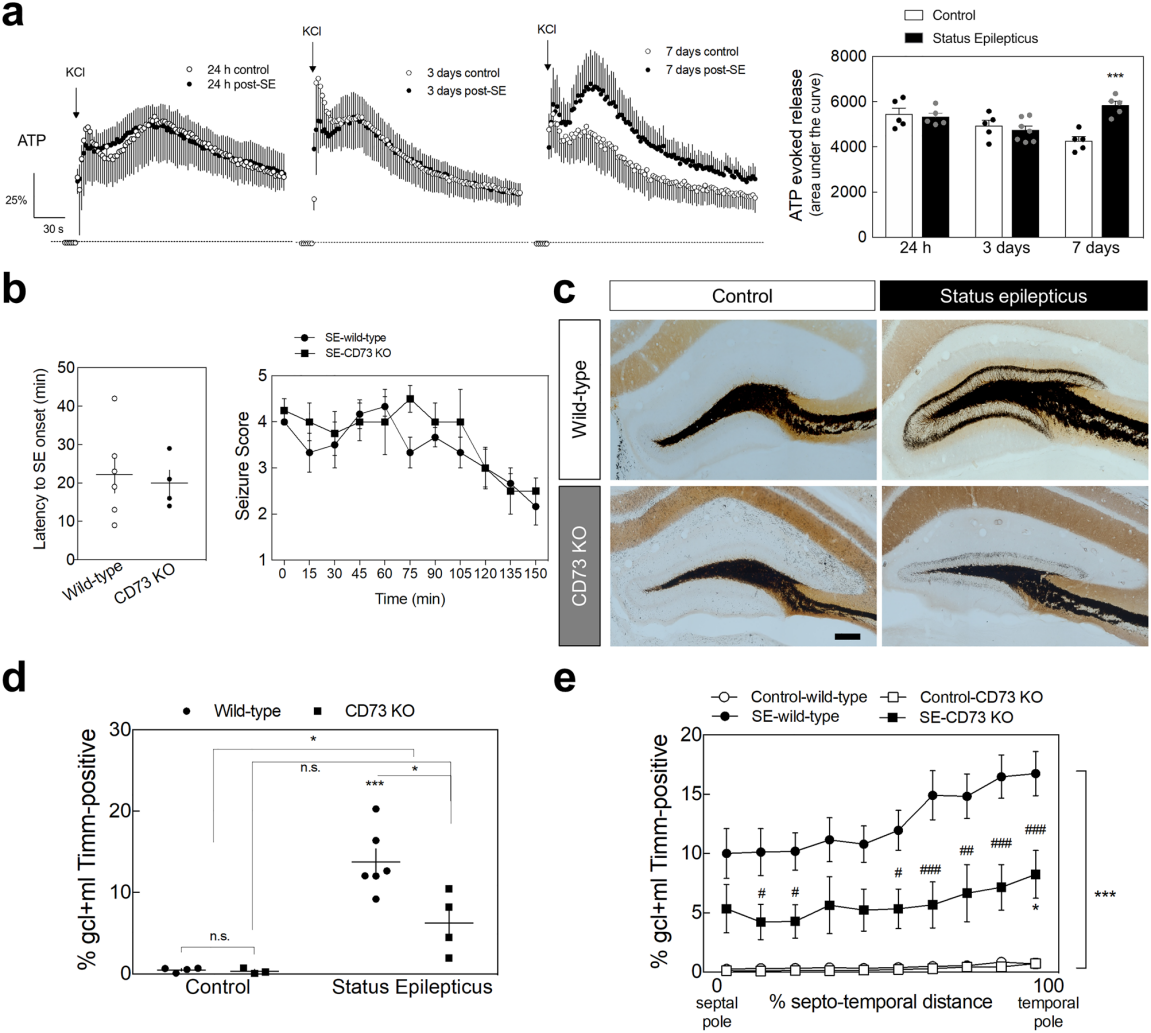
ATP-derived adenosine contributes to status epilepticus-induced hippocampal mossy fiber sprouting. (**a**) The evoked presynaptic release of ATP induced by a K^+^-induced depolarization (KCl 30 mM) of purified hippocampal nerve terminals prepared from animals that experienced status epilepticus (SE) was analyzed at 24 h, 3 days and 7 days upon SE induction. Only animals reaching at least stage 4 seizures were considered. Convulsions were suppressed with diazepam (10 mg/kg, *i.p.*) 2 h after the occurrence of the first stage 4 seizure. While at 24 h and 3 days after SE induction, it was not observed any significant differences, the evoked release of ATP from hippocampal synaptosomes prepared 7 days after SE was significantly higher *vs.* control rats. Data are mean ± SEM of the percentage of modification in extracellular levels of ATP (time-course graphs) or of the area under the curve (histogram). Control (24 h), *n* = 5; SE (24 h), *n* = 5; Control (3 days), *n* = 5; SE (3 days), *n* = 7; Control (7 days), *n* = 5; SE (7 days), *n* = 5. The data are mean ± SEM of the percentage of modification in extracellular levels of ATP relative to basal levels. ***P < 0.001, one-way ANOVA with Sidak’s test. (**b**) Latency onset and seizure score during SE of wild-type mice and CD73-KO mice. Data are mean ± SEM. (**c**) Wild-type mice that had experienced SE displayed a dense Timm staining in the inner molecular layer of the dentate gyrus 105 days later, whereas in CD73-KO mice SE induced a significantly lower Timm staining, as depicted in the representative images (scale bar, 300µm) and quantitatively summarized in the (**d**) upper histogram. (**e**) The lower SE-induced mossy fiber sprouting in CD73-KO mice was observed along the entire septotemporal axis of the DG. The data are mean ± SEM of the percentage of the granule cell layer (GCL) plus molecular layer (ML) that displayed Timm staining. Control-wild-type, *n* = 4; Control-CD73 KO, *n* = 3; SE-wild-type, *n* = 6; SE-CD73 KO, *n* = 4. *P < 0.05 and ***P < 0.001 for SE *vs.* Control either in wild-type or in CD73-KO mice; ^#^*P* < 0.05, ^##^*P* < 0.01, ^###^*P* < 0.001 for CD73-KO *vs.* wild-type mice either experiencing SE or not; two-way ANOVA with Sidak’s test.

ATP is preferentially released at high-frequency stimulations and A_2A_Rs are mainly activated by ATP-derived adenosine through the catabolism of extracellular ATP involving ecto-5’-nucleotidase (CD73) activity in MF terminals^17–19^. CD73, which catabolizes extracellular AMP into adenosine, is present in sprouted MF in epileptic rats^20^ and a SE-induced increased activity of CD73 has been reported in the pilocarpine rat model of temporal lobe epilepsy^49,50^. CD73 density is also enhanced in the inner molecular layer of the DG from human temporal lobe epilepsy patients^51^. We now measured a sustained increase of the evoked release of ATP from hippocampal nerve terminals prepared from rats 7 days after pilocarpine-induced SE in comparison with control rats, but not at 24 h or 3 days post-SE (Fig. 6a). This is coincident with the increased CD73 activity found in hippocampal nerve terminals from the same rat model of temporal lobe epilepsy^51^. This prompted the hypothesis that the adenosine triggering the observed A_2A_R’s contribution to SE-induced hippocampal MF sprouting derives from the extracellular catabolism of ATP in a CD73-dependent manner. To test this hypothesis, SE-induced MF sprouting was evaluated in CD73-KO mice. The latency to SE onset and the seizure score during SE were similar between wild-type and CD73-KO mice (Fig. 6b). In wild-type mice, SE induced a visible MF sprouting evaluated 105 days later (Fig. 6c). However, in CD73-KO mice, SE induced a significantly lower percentage of the granule cell layer plus molecular layer displaying Timm staining (*P* = 0.0129 for SE-wild-type *vs.* SE-CD73-KO), which was not statistically different from control (*P* = 0.1383 for SE-CD73-KO *vs.* Control-CD73-KO) (Fig. 6d). This significantly lower SE-induced MF sprouting in CD73-KO mice was also observed along the entire septotemporal axis (Fig. 6e), as observed with A_2A_R blockade.

Altogether, these results indicate that the adenosine triggering A_2A_R-mediated MF sprouting induced by SE is derived from the CD73-mediated extracellular catabolism of ATP.

## Discussion

The present study demonstrates that the overactivation of A_2A_Rs is a molecular mechanism underlying synaptic remodeling in the injured brain, namely SE-induced hippocampal MF sprouting. While A_2A_Rs did not contribute to the generation of recurrent excitatory inputs spontaneously developed in dentate granule cells of hippocampal organotypic slices (Fig. 2), the pharmacological blockade of A_2A_Rs significantly attenuated SE-induced hippocampal MF sprouting in a rat pilocarpine model (Fig. 3). This involves A_2A_Rs located in dentate granule cells since the knockdown of A_2A_Rs selectively in dentate granule cells reduced the density of SE-induced MF terminals in the inner molecular layer (Fig. 4). Moreover, the observation of an SE-induced increase in ATP release from hippocampal terminals, together with the prevention/attenuation of hippocampal MF sprouting in CD73-KO mice (Fig. 6), strongly suggests that A_2A_R-driven MF sprouting is triggered by extracellular ATP-derived adenosine.

We recently reported that A_2A_Rs control neuronal polarization and axon formation of cortical projection neurons during embryogenesis^19^. In the present study, we found that A_2A_Rs also control axon formation in developing rat hippocampal neurons (Fig. 1). However, in hippocampal neurons, A_2A_Rs did not control the formation of the “normal” axon; instead, the pharmacological activation of A_2A_Rs induced the formation of aberrant secondary axons (Fig. 1). Moreover, we now demonstrated the involvement of A_2A_Rs in SE-induced hippocampal MF sprouting (Fig. 3). This contribution of A_2A_Rs to MF sprouting was only observed in pathological conditions, *i.e.* in SE-induced MF sprouting, but not in the recurrent excitatory inputs that develop spontaneously in hippocampal organotypic slices (Fig. 2). This indicates that spontaneous MF recurrent sprouting in hippocampal organotypic slices is mechanistically distinct from epilepsy-related hippocampal MF sprouting and argues that an increased activity of A_2A_Rs after SE^23^ is required to trigger the abnormal axonal sprouting after SE. The observation that the knockdown of A_2A_Rs in granule cells was sufficient to reduce MF sprouting (Fig. 4), together with the ability of A_2A_Rs’ activation to induce the formation of abnormal secondary axons in cultured rat hippocampal neurons (Fig. 1) strongly suggests that A_2A_Rs contribute to hippocampal MF sprouting by directly promoting axon formation/outgrowth selectively during pathological conditions in the adult brain.

This newly described A_2A_R-mediated mechanism seems intrinsic to neurons rather than involving astrogliosis and inflammation, which are also characteristic features of epilepsy^52,53^ that are associated with the development of seizures^54^ and with the ability to contribute to synaptic remodeling^55^. Although both astrogliosis and neuroinflammation are also controlled by A_2A_Rs^17^, it was previously reported that blocking A_2A_Rs abrogates SE-induced neurodegeneration without affecting astrogliosis^24^ and it was now observed that the selective elimination of A_2A_Rs in hippocampal neurons was sufficient to prevent SE-induced MF sprouting. Thus, the proposed conclusion that A_2A_R-mediated control of SE-induced MF sprouting seems to involve an intrinsic neuronal remodeling by the adenosine neuromodulation system, seems the most parsimonious explanation, which may entail a direct regulation of cytoskeleton, as observed in developing rat cortical neurons^56^. Nevertheless, MF sprouting has been also shown to be correlated with cell loss, although it is not mandatory^57^. In particular, the extension of hippocampal MF sprouting has been correlated with the loss of hilar inhibitory interneurons and mossy cells^57^ found both in experimental models and in patients with temporal lobe epilepsy^58–63^. Another study also reported a positive correlation between MF sprouting and cell loss both CA3 and CA1^64^. As mentioned, the antagonism of A_2A_Rs prevents SE-induced neurodegeneration^23^. This has been associated to an up-regulation of neuronal/synaptic A_2A_R-driven increase in excitatory drive leading to calpain-mediated neurodegeneration^23^. More recently, it was observed an absence of neurodegeneration upon SE in CD73-KO mice^24^. The involvement of A_2A_Rs and of CD73 in hilar neurons death was not detailed^23,24^. Nevertheless, there is the possibility of an additional indirect contribution of the CD73-A_2A_R axis to hippocampal MF sprouting by controlling hippocampal cell damage.

This study determined that the contribution of A_2A_Rs to SE-induced hippocampal MF sprouting here identified entails, at least in part, A_2A_Rs located in dentate granule cells generated before SE (Fig. 4). But newly born cells also contribute to the abnormal retrodirective innervation of the inner molecular layer^38,41,42^. There are evidences suggesting for an inhibitory action of A_2A_Rs in neurogenesis in postnatal and adult rodent brain, in particular in cell proliferation^44^. A_2A_Rs were shown to contribute to the transient decrease in cell proliferation from the subgranular zone upon oxygen–glucose deprivation, most likely by contributing to the damage of immature cells^21^. On the other hand, it was recently reported that the activation of A_2A_Rs prevents the reduction of newborn dentate granule cells as a consequence of hearing loss induced by noise exposure^43^. Here, we observed that the pharmacological blockade of A_2A_R per se did not alter adult-born cell generation from the subgranular zone (Fig. 5). However, we found a statistically significant interaction between SCH58261 and SE in cell proliferation, but not in their positioning within the granule cell layer (Fig. 5). This suggests that SE-induced dentate granule cell proliferation, but not their migration, entails, either directly or indirectly, the activation of A_2A_Rs. Hence, A_2A_Rs may further contribute and sustain MF sprouting by promoting the generation of adult born cells induced by epileptogenic injury. The newborn cells only contribute to the abnormal innervation of the inner molecular layer upon their full maturation within 4 weeks^41,42^, although immature granule cells are able to extend axons to CA3^65,66^. Thus, it will be now interesting to elucidate if A_2A_Rs contribute to MF formation/outgrowth independently of the postsynaptic target or if A_2A_Rs are only involved in the growth of abnormal retrodirective axons. In addition, it is worth noting that we recently showed that A_2A_Rs also play a key role in GABAergic synapses stabilization^20^. Although we cannot yet extend this role of A_2A_Rs to the stabilization of MF synapses, we do not discard also an eventual postsynaptic effect of A_2A_Rs in the stabilization/formation of MF contacts. Hence, our findings leave open the possibility that A_2A_Rs may control circuit remodeling in the adult brain not only by the presynaptic regulation of axogenesis, either directly or indirectly, but also through the reactivation of their ability to control synapse stabilization/maturation.

The gain of function of neuronal A_2A_Rs after SE to control MF sprouting provides a functional correlate between the previously reported increased density of A_2A_Rs in nerve terminals after SE^22^ and the increased density of CD73, which has been proposed to be a marker of synaptic remodeling after SE^51^. The present observations that the synaptic release of ATP is increased after SE (Fig. 6a) and that the knockout of CD73 dampens SE-induced MF sprouting (Fig. 6b–e) allows concluding that noxious brain conditions such as SE trigger an upregulation of the whole ATP-CD73-A_2A_R axis to control MF sprouting. Although this bolstered ATP-CD73-A_2A_R axis seems to be recurrently engaged in the control of brain and synaptic dysfunction in different animal models of brain diseases^24,67,68^, the molecular mechanisms engaged by A_2A_Rs to tinker with synaptic remodeling are still not defined. The control of synaptogenesis during development involves an A_2A_R-mediated control of cAMP levels in synaptic terminals^20^, but the A_2A_R-mediated control of synaptotoxicity after noxious stimuli seems instead to involve p38-mediated signaling^69^. A possible A_2A_R-mediated control of BDNF and TrkB function^70^ is also grounded on conflicting evidence: although several evidences point towards an involvement of an up-regulation of BDNF in triggering this initial branching^9–11,13,71^, this does not seem sufficient per se to induce MF sprouting^12,14^. Furthermore, both the A_2A_R-mediated control of synaptogenesis during development as well as the A_2A_R-driven axonal elongation in cultured cortical neurons were not modified by the presence of a BDNF scavenger^20,56^. Clearly, the transducing pathways operated by these pleiotropic A_2A_Rs to control MF sprouting still remains to be unraveled.

Since the selected model to induce a pathological-like sprouting process in the adult brain is also relevant to model epileptogenesis, it is of interest to tentatively frame the present findings in the context of epilepsy. Adenosine has been for long known as an anti-convulsant^72^ via A_1_ receptors (A_1_R) activation^73^, which are mostly activated by adenosine released per se, depressing basal excitatory transmission^74^. However, during epileptogenesis there is a down-regulation of A_1_Rs indicated by both a decreased density of A_1_Rs in excitatory synapses in animal models of epilepsy^22^ and a reduction of ambient levels of adenosine, mostly driven by an increased activity of adenosine kinase^75,76^. In contrast, A_2A_Rs become prominent at high-frequencies stimulations, activated by adenosine originated from the CD73-dependent extracellular catabolism of ATP that is prominently released at high-frequencies^17,18,24^. Moreover, it was reported an increased density of A_2A_Rs in different animal models of epilepsy^22,77,78^ and in patients with epilepsy^79^, together with the prominent synaptic release of ATP at seizure-like firing patterns^17,80–82^ and an increase of the density and activity of CD73^24,49–51,79^, which supports a gain of function of A_2A_Rs during epileptogenesis. Indeed, as mentioned, A_2A_Rs tether SE-induced increase in neuronal excitability to hippocampal neurodegeneration^23^, an effect that is dependent on CD73 ^24^. Moreover, although we have not directed the present study to investigate this question, it is interesting to note that several studies demonstrated anti-convulsant effects upon inhibition of A_2A_Rs^83–86^. More importantly, the ability of the pharmacological inhibition of A_2A_Rs to mostly prevent the progressive seizure severity in kindling protocols^23,25,86^ and to arrest long-term deleterious consequences of early-life convulsions^87^, strongly indicate a relevant contribution of A_2A_Rs to epileptogenesis. This is in accordance with the presently concluded ability of A_2A_Rs to contribute to the circuit remodeling characteristic of epileptogenesis. However, despite the evidence indicating that MF sprouting forms recurrent excitatory inputs^3,4,88–92^, the link between MF sprouting and progressive hyperexcitability and seizure severity is still a working hypothesis. It may be pro-epileptogenic, homeostatic or an epiphenomenon^2,93^ or may be contributing to epilepsy comorbidities rather than seizure severity^94^. Interestingly, A_2A_Rs and CD73 also contribute to some of the comorbidities associated to epilepsy^23,24^. Further studies are now needed to unravel the functional consequences of the involvement of A_2A_Rs and CD73 in epilepsy-related circuit remodeling here identified.

Overall, the findings presented shown that A_2A_Rs contribute to epilepsy-related circuit remodeling, which may involve the reactivation of the ability of A_2A_Rs to control axon formation and outgrowth during development^19^. This posits A_2A_Rs as potential targets to manipulate aberrant synaptic remodeling during epileptogenesis, which may be extended to other disorders displaying also circuit alterations such as neuropsychiatric disorders affecting adults and prevalent on ageing.

## Methods

### Animals

Male Sprague–Dawley rats (Charles River Laboratories, Barcelona, Spain), global CD73-knockout (KO)^95^ and male wild-type and CD73-KO mice (6, 7 weeks old) were used. CD73-KO mice from a C57BL\6 background were generated and crossbred as previously described^19^. Animals were housed in groups of two (rats) or four (mice) *per* cage. Female pregnant Wistar rats (E18) were used for preparation of rat hippocampal cultures. P5-P7 male C57BL/6 mice were used for preparation of organotypic hippocampal slice cultures. Animals were kept in ventilated cages with food and water available ad libitum under a 12 h light/dark cycle in a thermoregulated environment. All animal procedures followed the ARRIVE guidelines and the European legislation (European directive 2010/63/EU) as approved by the Institutional Animal Care and Use Committees of the Center for Neuroscience and Cell Biology and Instituto de Neurociencias CSIC-UMH (Alicante, Spain), and the Portuguese Law and Ordinance, approved by the government agency *Direção Geral de Alimentação e Veterinária* (0421/000/000/2013).

### Hippocampal neuronal cultures, in vitro electroporation and drug treatments

Rat hippocampal neuronal cultures were prepared from Wistar E18 rat embryos as previously described^69^ at a cell density of 1000 cells/cm^2^. In vitro electroporation of dissociated cells with validated shRNA-A_2A_R and a non-targeting control (shRNA-Control) encoding EGFP (^31^; see Supplementary Figs. 1, 2) was performed using Ingenio Electroporation kit (Mirus Biotech®; Cat#MIR 50118) and Nucleofector® IIb device (Amaxa Biosystems) as previously described^19^. Two hours after plating, CGS21680 and SCH58261 (Tocris) were added. Cells were kept at 37 °C in a 5% CO_2_ atmosphere until use for immunochemical processing at 3 days in vitro (DIV3). The concentrations of A_2A_R ligands used were previously shown to be selective and supra-maximal for A_2A_Rs in hippocampal preparations, either cell cultures or slices^18,69^.

### Immunocytochemistry

Immunocytochemical analysis was performed as previously described^19^ using as primary antibodies rabbit anti-βIII-tubulin (1:1000; Abcam, Cat#ab18207; RRID:AB_444319) and SMI31 (1:1500; BioLegend, Cat#801601; RRID: AB_2564641) or mouse anti-A_2A_Rs (1:200; Milipore, Cat#05–717; RRID:AB_11213750) and the respective AlexaFluor-conjugated secondary antibodies (1:1000; ThermoFisher Scientific; Cat#A31572, RRID:AB_162543; Cat#A21202, RRID:AB_141607; Cat#A32728, RRID:AB_2633277). Actin cytoskeleton was labelled using Alexa Fluor-633-conjugated phalloidin (1:100; Cat#A22284). Images were acquired using Zeiss Axio Imager Z2 fluorescence microscope or LSM 710 confocal microscope with Zen Blue/Black 2012 software.

### Hippocampal slice cultures

Hippocampal slice cultures were performed using the interface method^96^. Briefly, brains from P5-P7 C57Bl/6 mice were extracted in ice-cold high-sucrose medium containing (in mM): 233 sucrose, 26 NaHCO_3_, 10 glucose, 4 KCl, 5 MgSO_4_, 1 CaCl_2_. Hippocampi were dissected and cut in 400 µm-thick transversal slices, transferred to semipermeable membranes with MEM culture medium (Invitrogen) containing 20% horse serum, 7.5 mM glucose, 1.5 mM MgSO_4_, 1% insulin and 0.0012% ascorbic acid, and kept in an incubator at 35.5 °C for 7 days before recording. Slices were incubated with SCH58261 (100 nM) or vehicle in culture medium from day 0 in culture until two days prior recording.

### Electrophysiological recordings in organotypic hippocampal slices

Semipermeable membranes containing cultured hippocampal slices were transferred to pre-warmed (32 °C) carbogen-saturated artificial cerebrospinal fluid (ACSF, in mM: 124 NaCl, 26 NaHCO_3_, 10 glucose, 3 KCl, 1.25 NaH_2_PO_4_, 1 MgSO_4_ and 2 CaCl_2_) and maintained for at least 1 h prior recording. Recordings of DG granule cells were performed in voltage-clamp configuration using 2, 3 MΩ resistance borosilicate glass pipettes filled with internal solution containing (in mM): 130 CsMeSO_3_, 4 NaCl, 10 HEPES, 10 tetraethylammonium, 1 EGTA, 5 QX314, 2 ATP and 0.5 GTP. Picrotoxin (50 μM; Tocris) was applied to the ACSF to block GABA_A_ receptors and the membrane potential was clamped at − 60 mV. An ACSF-filled monopolar electrode was placed in the hilus and the stimulation of MF was performed every 20 s to record synaptic responses onto granule cells.

### Pilocarpine SE model

Pilocarpine hydrochloride (Sigma) was administered (*i.p.*) to male Sprague–Dawley rats or wild-type or CD73-KO mice to induce SE, characterized by continual stage 3, stage 4 and stage 5 seizures according to the Racine’s standard classification^97^. Scopolamine methylbromide (2 mg/kg, *i.p.*; Sigma) was injected 30 min before pilocarpine to minimize peripheral muscarinic cholinergic effects. Convulsions were suppressed with diazepam (10 mg/kg, *i.p.*) 2 h (for rats) or 2.5 h (for mice) after the onset of SE, defined by the occurrence of the first stage 4 seizure. Only animals reaching at least stage 4 seizures were considered in this study. Control animals received the same treatments as experimental animals except that they were given saline in place of pilocarpine. For the evaluation of the impact of SCH58261 in hippocampal MF sprouting and cell proliferation, rats were injected with 380 mg/kg of pilocarpine. In rats previously subjected to stereotaxic lentiviral injection, SE was induced 10 days later with 350 mg/kg of pilocarpine. In wild-type mice or CD73-KO mice, SE was induced with 340 mg/kg of pilocarpine.

### SCH58261 and BrdU administration in vivo

Rats that had experienced SE reaching at least stage 4 were paired based on seizure scale and assigned into two groups. One group was daily *i.p.* injected with vehicle (0.2% Tween-20 in saline) and the other with the selective A_2A_R antagonist SCH58261, which displays no binding in A_2A_R-KO mice^98^, with an efficacious dose of 0.1 mg/kg^32,67^ that was previously pharmacokinetically validated^99^, starting at 10 h after the onset of SE for 42 days. Control animals were also daily injected with vehicle or SCH58261.

BrdU (ThermoFisher Scientific) was slowly and completely dissolved in warmed (40-50 °C) sterile saline solution at 10 mg/mL and immediately used. Rats were injected with BrdU (50 mg/kg; *i.p.*) at the 1st (4 injections), 7th (4 injections) and 8th (2 injections) days post-SE with 2 h intervals between injections, based in a previous study^38^.

### Stereotaxic lentiviral injection

After the anesthesia of rats with ketamine-xylazine (128 mg/kg, *i.p.* and 14 mg/kg, *i.p.*, respectively), lentiviral vectors (1.5 μL at 300 µg of p24 antigen *per* mL) encoding shRNA-Control or shRNA-A_2A_R and enhanced green fluorescent protein (EGFP) under the H1 and PGK promoters respectively, previously validated and detailed (^31,32^;see also Supplementary Figs. 1, 2), were stereotaxically injected into the DG at a rate of 0.25 μL/min with an automatic injector (Stoelting) at the following coordinates: Anterior–Posterior (AP) − 3.2 mm, Medial–Lateral (ML) ± 1.2 mm, Dorsal–Ventral (DV) − 4.1 mm; AP − 4.0 mm, ML ± 2.0 mm, DV − 3.7 mm; and AP − 5.0 mm, ML ± 2.9 mm, DV − 3.8 mm^99^. This lentiviral vector was shown to ensure a long-lasting knockdown of A_2A_Rs^31,32^. Half of the animals were administered with shRNA-A_2A_R in the left hemisphere and shRNA-Control in the right hemisphere, whereas the other half received shRNA-A_2A_R in the right hemisphere and shRNA-Control in the left hemisphere. The injection needle was withdrawn 2 min after the end of each injection. Rats were kept in their home cages for 10 days for recovery before being further manipulated.

### Timm staining

The animals were deeply anesthetized with an overdose of sodium pentobarbital and perfused for 3 min with 0.9% NaCl, 7.5 min with 0.37% Na_2_S, 1.5 min with 0.9% NaCl and 45 min with 4% paraformaldheyde in 0.1 M phosphate-buffered saline (PBS). After perfusion, the brains were collected and post-fixed overnight. They were then transferred into a 30% sucrose solution diluted in PBS for 1 day (for mice) or 2, 3 days (for rats) and sectioned into 40 μm coronal sections using a cryostat (Leica CM3050S) at − 22 °C for histological analysis. A 1-in-6 series of brain sections from the septotemporal length of the hippocampus of each animal (240 μm apart, 10 or 12 sections in total) were mounted onto gelatin-coated glass slides. After rehydration, the sections were kept in a mixed solution composed of 120 mL of 50% gum arabic (w/v), 60 mL of 5.67% hydroquinone (w/v), 20 mL of 2 M citrate buffer, and 1 mL of 17% silver nitrate (w/v) for 45 min in the dark. After three 10-min washes in water, sections were dehydrated in 50, 70, 95, and 100% alcohol, cleared in xylene and mounted with DPX mounting medium (Sigma). Entire sections composed of multiple image tiles including the entire DG were acquired using a Zeiss Axio Imager Z2 upright fluorescence microscope equipped with a CCD color digital camera (Axiocam HRc) and an objective EC Plan-Neofluar 10x/0.3 Pol M27 (for rat sections) or Plan-Apochromat 20x/0.8 M27 (for mouse sections) using the Zen Blue 2012 software (Zeiss).

### Immunohistochemistry

The free-floating coronal brain Sects. (40 μm thick) were permeabilized and blocked in PBS (Sigma) containing 0.3% Triton X-100 (Fisher BioReagents) and 10% normal donkey serum (Merck Millipore) for 2 h at room temperature. For bromodeoxyuridine (BrdU) staining, slices were treated with 2 M HCl for 20 min at 37 °C followed by washing in borate buffer (0.1 M, pH 8.5) and in PBS (10 min, 3x) prior to permeabilization and blocking. Sections were then incubated overnight at 4 °C with the following primary antibodies in blocking solution: rat anti-BrdU (1:200; AbD Serotec, Cat#OBT0030G, RRID: AB_609567), rabbit anti-GFAP (1:1000; Milipore, Cat#AB5804, RRID:AB_305124) and mouse anti-NeuN (1:400; Milipore, Cat#MAB377, RRID:AB_2298772) or rabbit anti-synaptoporin (1:500, Synaptic Systems, Cat#102002, RRID: AB_312851). Slices were then washed in incubation solution (10 min, 5 times) and exposed to the respective fluorescent Alexa-Fluor®-conjugated secondary antibodies (1:500, ThermoFisher Scientific; Cat#A-21208, RRID: AB_2535794; Cat#A-31572, RRID: AB_162543, and Cat#A-31571, RRID: AB_162542) overnight at 4 °C. After washing in PBS (10 min, 3 times), sections were mounted on gelatin-coated glass slides using Dako fluorescent mounting medium (Dako). Images were acquired on a Zeiss confocal laser scanning LSM 710 microscope with a 40x/1.4 numerical aperture oil objective using the Zen Black 2012 software (Zeiss). For BrdU, NeuN and GFAP triple-immunostained slices, entire sections composed of multiple image tiles including the entire DG were acquired. For synaptoporin, NeuN and EGFP sections, vertical z-stacks were captured with a 2 μm step width.

### ATP release from rat hippocampal synaptosomes.

Rat hippocampal synaptosomes were prepared as previously described^67^ at 24 h, 3 days and 7 days post-SE. The release of ATP was measured on-line using the luciferin-luciferase assay as previously^67^. The light emitted was recorded every 2 s. After 60 s measuring basal ATP outflow, the evoked release of ATP was triggered with 30 mM of KCl (isomolar substitution of NaCl in the Krebs-HEPES solution) and further recorded for 200 s after the chemical stimulation. The evoked release of ATP was calculated by integration of the area of the peak upon subtraction of the estimated basal ATP outflow.

### Experimental design and statistical analysis

#### Morphometric analysis of axons

Axons in cultured rat hippocampal neurons were identified by immunolabelling with SMI-31 antibody (axonal marker) together with βIII-tubulin (neuronal marker) and GFP (electroporated cells, when applicable) at DIV3. SMI-31 does not label MAP2^+^-neuritic/dendritic processes^19,101^. SMI31^+^-structures displayed a thinner and > 50 µm long morphology characteristic of axons. Neurolucida software (MBF Biosciences) was used for image processing and morphometric analysis. The quantification of axonal length was derived from the β-III-tubulin labelling to ensure tracking the entire process. All measurements were performed in a blind-manner. A minimum of 100 cells *per* pharmacological condition *per* culture were quantified. A minimum of 25 electroporated GFP^+^-cells *per* condition *per* culture were analyzed.

#### MF sprouting analysis by Timm staining

MF sprouting was analyzed as previously described^102,103^. The extent of MF sprouting was assessed by estimating the fraction of the total volume of the granule cell layer plus molecular layer that was Timm^+^. To determine the Timm^+^ area in each section, a contour was drawn first around the granule cell layer plus inner molecular layer (Contour 1). The color images were then converted to black and white images to determine the Timm^+^ area within the Contour 1 by adjusting a darkness threshold tool. A Contour 2 was drawn to measure total granule cell layer + molecular layer area. The extent of MF sprouting in each section was then calculated as the % of Timm^+^-area within Contour 1 over the total granule cell layer + molecular layer area (Contour 2). For the entire hippocampus, the areas measured in each section were multiplied by six (1 every 6 sections were used) and 40 μm, and summed, to estimate volumes. Imaging processing and analysis was performed with ImageJ software.

#### MF sprouting analysis by synaptoporin puncta density in lentiviral-infected DG

MF sprouting was assessed by synaptoporin immunoreactivity, which reliably labels the terminals of MF^32–34^. For the analysis, the granule cell layer defined by NeuN immunoreactivity was divided into 2 regions: region 1 with higher EGFP^+^-cell density (ED1) and region 2 with relatively lower EGFP^+^-cell density (ED2). The EGFP^+^-cell density was calculated by dividing the EGFP^+^-cell number by the granule cell area for each region. The corresponding inner molecular layer was then also separated into 2 regions, region I and region II, and the synaptoporin puncta density in each region (PDI and PDII) was calculated. Puncta were defined as synaptoporin immunoreactivity with an area larger than 0.2 μm^2^. The correlation between EGFP^+^-cells density, either for shRNA-Control or shRNA-A_2A_R, and synaptoporin puncta density in the inner molecular layer, was determined using following Δsprouting ratio:

$$ \Delta {\text{sprouting}}\;{\text{ratio}} = \frac{{\frac{{\text{PDI - PDII}}}{{{\text{PD2}}}}}}{{\frac{{\text{ED1 - ED2}}}{{{\text{ED2}}}}}} $$

The Δsprouting ratio for each DG of an individual animal was calculated by averaging all the Δsprouting ratios determined from all the images acquired from that infected DG. The EGFP^+^-cells were quantified from z-stack images and synaptoporin puncta density from the respective confocal maximum projections. This analysis was designed to avoid bias arising from different infection levels in different regions of the granule cell layer and in different regions of the septo-temporal axis, and different levels of synaptoporin immunoreactivity in the inner molecular layer across the orthogonal axis of the DG, providing an internal control within each DG in addition to the internal control in each animal.

#### BrdU^+^-cell proliferation and migration

All BrdU^+^-cells located within the granule cell layer in every 12th coronal section were counted. The sum of counted cell number was then multiplied by 12 to obtain an estimate of the total number of BrdU^+^- cells in the granule cell layer *per* DG. To quantify the migration of the BrdU^+^-cells, granule cell layers were divided into the inner one-third and outer two-thirds and the relative percentage of BrdU^+^-cells located in the inner one-third and in the outer two-thirds of the granule cell layer was quantified.

### Statistical analysis

All data are presented as mean ± standard error of the mean (SEM) of n experiments or median with interquartile interval and minimum and maximum values. Fold changes and Δsprouting ratio were analyzed by one-sample student’s *t*-test *vs*. an hypothetical value of 1 or 0, respectively. Differences between two groups were analyzed using a two-tailed unpaired student’s *t*-test. A paired student’s *t*-test was applied for the comparison of Δsprouting ratio between shRNA-A_2A_R and shRNA-Control. Differences between more than two groups were analyzed by one-way ANOVA with Dunnett’s *post-hoc* test or Sidak’s multiple comparison test. The percentage of neurons displaying a different number of axons, the impact of CGS28160 in cells electroporated with shRNA-Control or shRNA-A_2A_R regarding the number of axons *per* neuron, the % of granule cell layer + molecular layer with Timm staining, either total or through the septo-temporal axis, and the cell proliferation and migration were analyzed by two-way ANOVA. Where significant interactions were detected, *post-hoc* comparisons were performed using Sidak’s test. A familywise 95% confidence level (*P* < 0.05) was applied. All data processing and analyses were performed using Prism 6.0 software (GraphPad).

### Animal use ethical approval

All the procedures in animals were approved by the Institutional Animal Care and Use Committees of the Center for Neuroscience and Cell Biology and Instituto de Neurociencias CSIC-UMH (Alicante, Spain), in accordance with ARRIVE guidelines and European legislation (European directive 2010/63/EU) and the Portuguese Law and Ordinance, approved by the government agency *Direção Geral de Alimentação e Veterinária* (0421/000/000/2013).

## Supplementary Information

Supplementary Information.

## Data Availability

The data that support the findings of this study are available from the corresponding author upon reasonable request.
