# Supplementary material for: Adenosine A2A receptors control synaptic remodeling in the adult brain
Source: Sci Rep. 2022 Aug 29;12:14690. doi: 10.1038/s41598-022-18884-4 (PMC9424208; doi:10.1038/s41598-022-18884-4)
Supplement: Supplementary file 1 — Supplementary Information. [file 41598_2022_18884_MOESM1_ESM.pdf]

Adenosine A_{2A} receptors control synaptic remodeling in the adult brain

Xinli Xu[1,2], Rui O Beleza[1], Francisco Q Gonçalves[1,2], Sergio Valbuena[3], Sofia Alçada-Morais[1,2], Nélio Gonçalves[1,2], Joana Magalhães[1,2], João M M Rocha[1,2], Sofia Ferreira[1,2], Ana S G Figueira[1], Juan Lerma[3], Rodrigo A Cunha[1,4], Ricardo J Rodrigues[1,2,&]* , Joana M. Marques[1,2,&]

[&]These authors contributed equally to the supervision of the work.

[1]CNC-Center for Neuroscience and Cell Biology, University of Coimbra, 3004-504 Coimbra, Portugal.

[2]Institute of Interdisciplinary Research, University of Coimbra, 3030-789 Coimbra, Portugal.

[3]Instituto de Neurociencias de Alicante, CSIC-UMH, 03550 San Juan de Alicante, España.

[4]Faculty of Medicine, University of Coimbra, 3004-504 Coimbra, Portugal

***Corresponding author**:

Ricardo J. Rodrigues; Center for Neuroscience and Cell Biology, Rua Larga, Faculdade de Medicina Polo I, Piso 1, 3004-504 Coimbra, Portugal; Email: ricardojrodrigues@gmail.com ; https://orcid.org/0000-0002-7631-743X

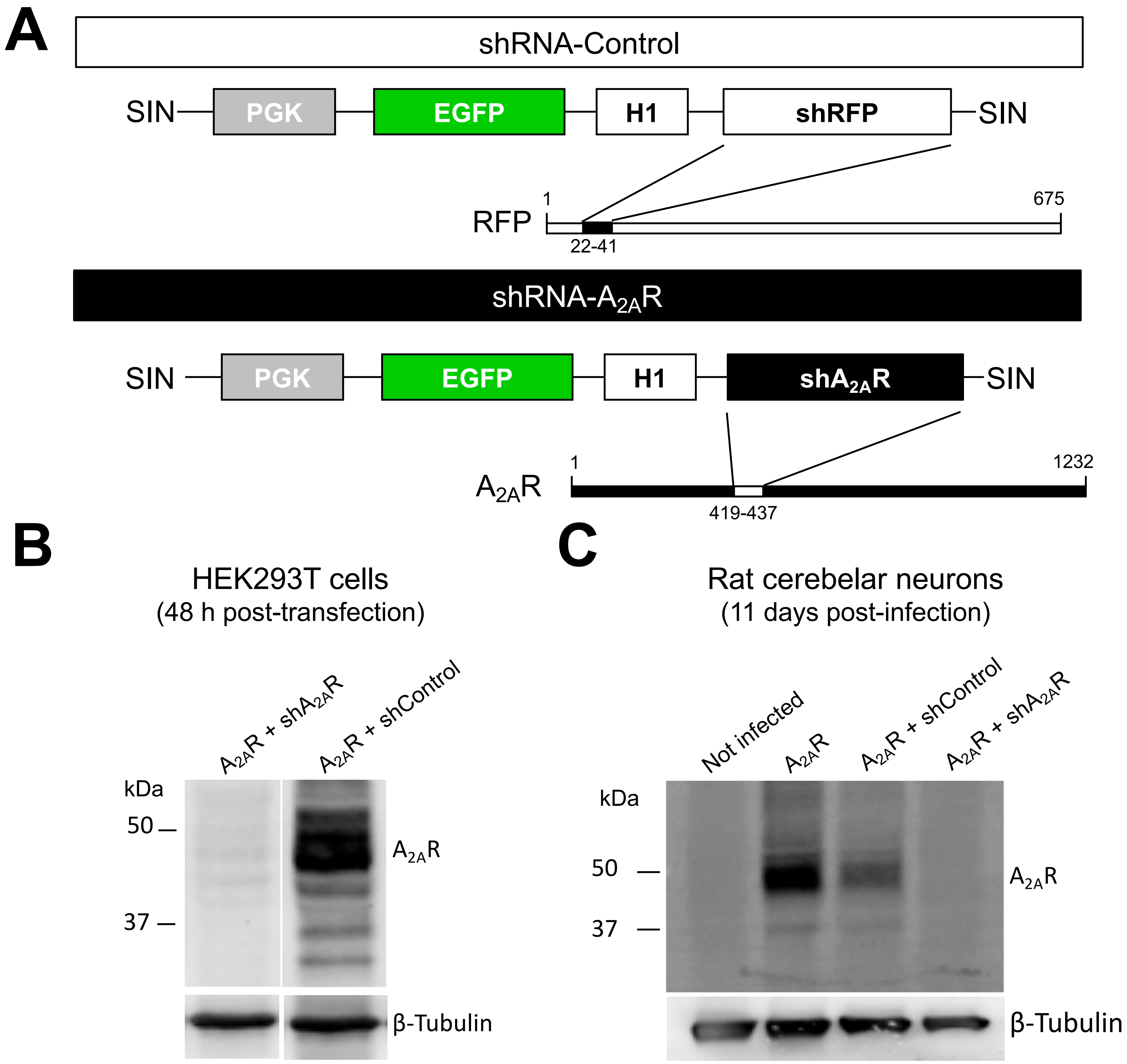

Supplementary Figure 1. (**A**) Schematic representation of the lentivector constructs used to downregulate $A_{2A}R$ (sh$A_{2A}R$, nt 419-437) and a mistargeted control (shRFP [red fluorescent protein], nt 22-41). The silencing small hairpin RNA cassettes are under control of the H1 promoter (pol III) and inserted in a lentivector containing the enhanced green fluorescent protein (EGFP) reporter gene under control of the phosphoglycerate kinase-1 (PGK-1) promoter. (**B**,**C**) Western blot analysis of HEK293T cells (**B**) and of rat cerebellar neurons (**C**) 48 h post-transfected and 11 days post-transduction, respectively, either with a lentivector encoding $A_{2A}R$ (GenBank BC110692) alone or in association with sh$A_{2A}R$ or the control (shControl=shRFP). Original blots are displayed in Supplementary Figure 2

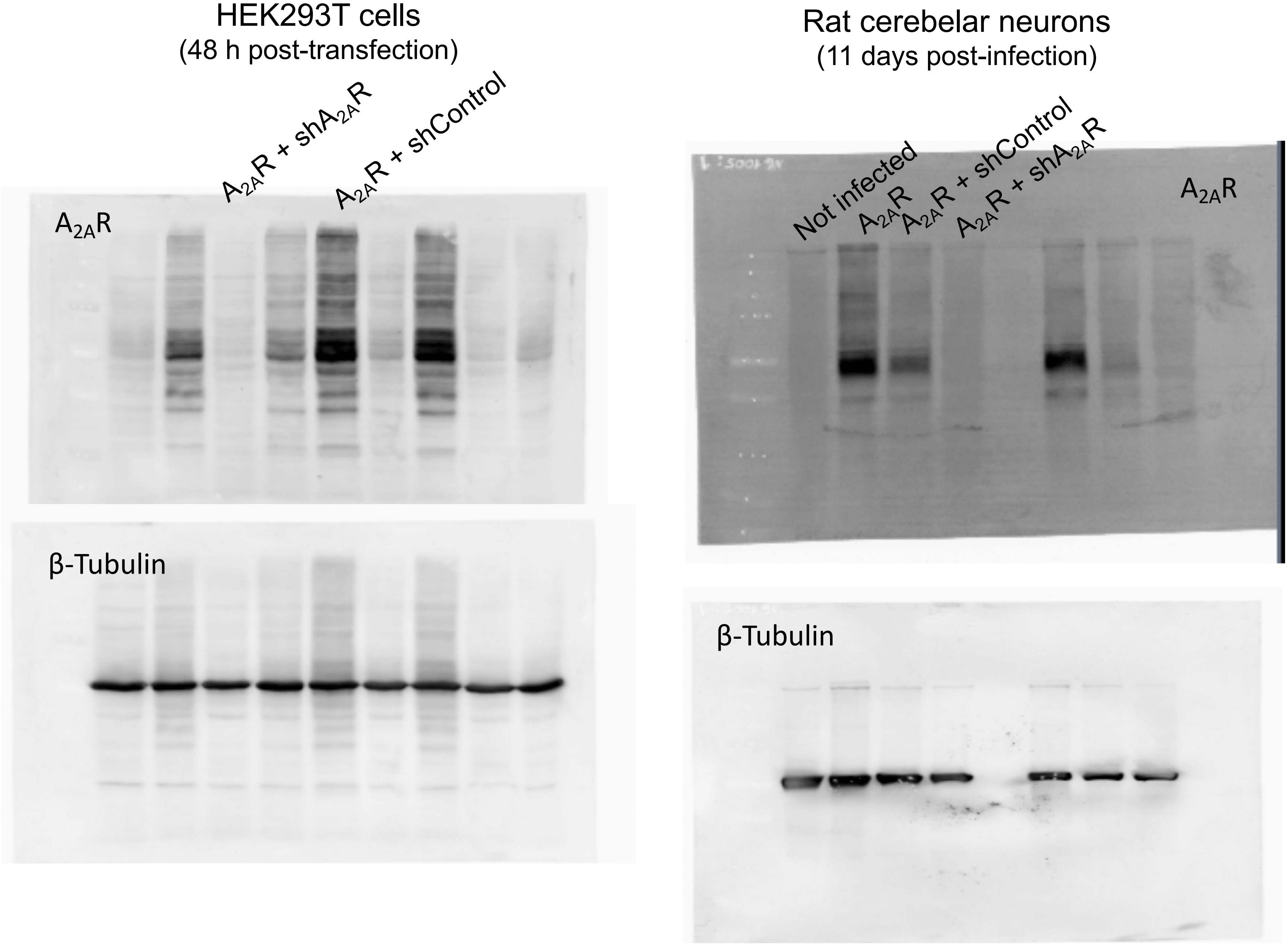

Supplementary Figure 2. Original Western blots displayed in Supplementary Figure 1.
